# The Role of Mandibular Third Molars on Lower Anterior Teeth Crowding and Relapse after Orthodontic Treatment: A Systematic Review

**DOI:** 10.1155/2014/615429

**Published:** 2014-04-30

**Authors:** Khalid H. Zawawi, Marcello Melis

**Affiliations:** ^1^Department of Orthodontics, Faculty of Dentistry, King Abdulaziz University, P.O. Box 80209, Jeddah 21589, Saudi Arabia; ^2^Craniofacial Pain Center, Tufts University School of Dental Medicine, One Kneeland Street, Boston, MA 02111, USA

## Abstract

*Aims*. To evaluate the role of third molars in the development of crowding or relapse after orthodontic treatment in the anterior segment of the dental arch. *Methods*. PubMed search of the literature was performed selecting all the articles relevant to the topic and limiting the studies to controlled trials on humans and written in English language. Systematic review was conducted according to the PRISMA (preferred reporting items for systematic reviews and meta-analyses) statement. *Results*. A total of 12 clinical studies were included in the review. A high risk of bias was found in most of the articles, either because the relative items assessed were inadequate or because they were unclearly described. The third molars were not correlated with more severe anterior tooth crowding in most of the studies. However, four of them described a different outcome. *Conclusion*. Definitive conclusions on the role of the third molars in the development of anterior tooth crowding cannot be drawn. A high risk of bias was found in most of the trials, and the outcomes were not consistent. However, most of the studies do not support a cause-and-effect relationship; therefore, third molar extraction to prevent anterior tooth crowding or postorthodontic relapse is not justified.

## 1. Introduction

In orthodontics, the most controversial role of the third molars is whether they can contribute to the development of malocclusion or relapse after orthodontic treatment, particularly in the anterior segment of the dental arch. While this subject has been discussed and presented in the literature, it is an issue that remains unresolved. It has been hypothesized that, while erupting, the tooth could transmit an anterior component of force down the dental arch concentrating in the areas of canines and incisors, which results in tooth rotation and misplacement [1, 2]. Based on such theory, Niedzielska suggested that, when a sufficient space is available for the eruption of the third molars, the tooth assumes a normal position in the dental arch and does not cause displacement of the other teeth; conversely, when the space is deficient, third molars may aggravate dental crowding [2]. However, several studies did not confirm these conclusions. Sidlauskas and Trakiniene [3] studied a group of ninety-one subjects with a mean age of 21 years. Registration of crowding was based on the mesiodistal width measurements of the teeth in relation to the length of the corresponding segment of the lower dental arch. No statistically significant differences were reported in terms of lower dental arch crowding between the groups with erupted, unerupted, and agenesis of third molars. They concluded that there is no evidence to implicate third molars as etiologic factors in the late lower dental arch crowding [3]. In addition, Karasawa et al. [4] evaluated three hundred subjects with a mean age of 20.4 years on the presence or absence of wisdom teeth and mandibular incisor crowding. They also found no statistically significant association between the presence of upper and/or lower third molars and anterior mandibular teeth crowding. Their conclusions stated that evidence on the role of third molars as etiologic factor in the late lower arch crowding is lacking, similarly to the ones of the previous study [4].

Harradine et al. [5] described analogous outcome in subjects who underwent orthodontic treatment. They examined the effect of third molar extraction on the development of anterior mandibular dental crowding in a randomized controlled study. The results show minimal difference between the groups (third molars extracted versus third molars nonextracted), and such difference was not statistically significant. It was also considered clinically nonsignificant; thus they concluded that removal of third molars to prevent or reduce late incisor crowding could not be justified [5].

In another study four groups of patients treated orthodontically were compared [6]. The groups consisted of subjects whose third permanent molar teeth had erupted into the mouth, were nonerupted, were extracted, and were congenitally absent. The irregularity index used to evaluate tooth misplacement was evaluated before, immediately after, and at least three years after orthodontic treatment; however, no difference was detected among the groups [6]. The role that mandibular third molars play in lower anterior crowding has been discussed and presented often in the orthodontic literature; nonetheless, it is an issue that remains unresolved. Several reviews were discussed this issue. Bishara [7] in his review acknowledged this controversial issue; however, he did not find any evidence to implicate these teeth as being the only or even the major etiologic factor in the posttreatment changes in incisor alignment. He also suggests that the only relationship between these two phenomena is that they occur at approximately the same time of development. The NHS Center for Reviews and Dissemination, University of York, UK, in their recommendation to the effectiveness and cost-effectiveness of prophylactic removal of third molars, suggested that there was only a weak association between retention and third molars and crowding. They also noted that the methodological quality of the literature reviews was generally poor and none of the reviews was systematic [8]. Mettes et al. [9] also showed no evidence to support nor refute regular prophylactic removal of asymptomatic impacted third molars in adults. They also found prophylactic removal of asymptomatic impacted third molars in adolescents neither reduces nor prevents late incisor crowding. However, since the review by Mettes et al. [9] almost ten years ago there have been several additional studies added to the literature. Therefore, in the light of the different results in the literature, the aim of this study was to review the articles published on the topic by following the preferred reporting items for systematic reviews and meta-analyses (PRISMA) statement [10], in order to clarify the role of mandibular third molars on lower anterior teeth crowding and relapse after orthodontic treatment.

## 2. Materials and Methods

### 2.1. Literature Search

Two reviewers (Marcello Melis and Khalid H. Zawawi) independently performed an electronic search of the literature using PubMed according to the following criteria. Key terms included in the search were* third molar/molars* and* wisdom tooth/teeth* on one side and* relapse, anterior crowding, alignment, post retention, anterior post retention, incisor relapse, *and* incisor crowding* on the other side. All key words were included both as medical subjects headings (MeSH) terms and text words. After combining the results, selections were limited to the English language and humans. After reading the titles and the abstracts selected, only original articles relevant to the topic were included in the review. Additionally, a manual search was carried out by examining the references of the included articles. Finally, only controlled trials of the articles collected were selected.

### 2.2. Quality Assessment of the Studies

To evaluate the quality of the studies included in the review the following items were assessed as described by Vos et al. [11]:

1. sequence generation and concealed allocation,
2. size and composition of the studied groups,
3. blinding of participants, clinicians, and investigators,
4. application of inclusion and exclusion criteria for subjects,
5. descriptions of loss to follow-up,
6. adequacy of statistical analysis.

Two reviewers (Marcello Melis and Khalid H. Zawawi) independently rated each study scoring it as “adequate” when the relative item was judged to be associated with a low risk of bias, “unclear” when lack of information on the relative item did not allow evaluating the risk of bias, and “inadequate” when the relative item was judged to be associate with high risk of bias.

Sequence generation and concealed allocation were considered adequate when the group assignment was randomized and the clinician was blind to such assignment. Size and composition of the studied groups were considered adequate when the size of the groups was approximately equal and age and gender were equally represented among the groups. Blinding of participants, clinicians, and investigators was considered adequate when at least the investigator who analyzed the results was blind to the condition of the subjects. However, blinding of participants and clinicians was considered impossible due to the features of the therapy (e.g., orthodontic treatment and dental extraction). Application of inclusion and exclusion criteria for subjects was considered adequate when they were properly described before the inclusion of the subjects. Descriptions of loss to follow-up were considered adequate when the number of withdrawals from the groups was clearly indicated. However, such evaluation was not applicable for cross-sectional studies, where loss to follow-up cannot occur. Adequacy of statistical analysis was considered adequate when all subjects included were analyzed and statistical tests were considered appropriate. An expert in statistics evaluated the appropriateness of statistical tests.

## 3. Results

A total of 96 articles were first found by combining the key words and limiting the studies to English and human. After examining the titles and the abstracts, twenty-six studies were identified, and 5 additional publications were added after the manual search of their references. Seven articles that were first selected by the title for further evaluation could not be found, mainly because the year of publication was very old. Thus, a total number of 12 controlled studies were included in the review (Table 1) [2, 3, 5, 6, 12–19].

The quality assessment of the selected studies revealed a high risk of bias in most of the articles, either because the relative items assessed were inadequate or because they were unclearly described. Generally, quality tended to improve in the more recent trials with respect to the oldest, but also more contemporary studies obtained a very low quality evaluation. The only article that scored “adequate” in all items was the one by Harradine et al. [5]; detailed assessment is shown in Table 2.

Of the 12 studies included in the review, 9 were longitudinal studies (3 of them prospective longitudinal and 6 of them retrospective longitudinal based on patients' records) and 3 were cross-sectional studies. The prospective longitudinal studies analyzed orthodontically treated or untreated subjects divided into different groups according to the presence or absence in occlusion of the third molars. The absence of the third molars in functional occlusion was due to extractions, impaction, or agenesis. One prospective longitudinal study did not divide the subjects into groups but used one side of the mandible as a control for the opposite side [15]. Subjects were followed up to detect the development of anterior tooth crowding for a defined period of time [2, 5, 15]. The retrospective longitudinal studies were carried out retrieving the clinical charts and dental models of orthodontically treated or untreated patients and assessing the association between the presence or absence of the third molars and the development of anterior tooth crowding [6, 13, 14, 16–18]. Cross-sectional studies analyzed orthodontically untreated subjects and correlated the presence or absence in occlusion of the third molars with the presence of anterior tooth crowding [3, 12, 19].

The presence of the third molars was not correlated with more severe anterior tooth crowding in most of the studies [3, 5, 6, 12, 14, 17–19]; however, some of them described a different outcome [2, 13, 15, 16]. Sheneman found more dental stability in orthodontically treated subjects with congenitally missing third molars when compared with subjects with either unerupted or erupted third molars at five-year follow-up [13]. Lindqvist and Thilander, after extracting the third molar on one side of the mandible, reported that the extraction side had a more favorable development of the dental arch in 70% of the cases [15]. Richardson reports that individuals whose third molars become impacted tend to have more tooth crowding [16]. Niedzielska [2] specified that subjects with retained third molars displayed increased tooth crowding in relation to Ganss ratio (the ratio between the third molar width and the retromolar space).

## 4. Discussion

Late crowding of the lower incisor teeth is frequently observed concurrently to the eruption of the third molars, inducing the clinicians to presume a cause-and-effect relationship between the two events. The hypothesis is that the mesial component of the forces created by the erupting third molars, transmitted through the dental arch, can create a mesial migration of the teeth culminating in the area of the incisors. The result is the loss of available space and crowding.

Most of the studies included in this systematic review did not support a cause-and-effect relationship between the eruption of the third molars and the development of anterior tooth crowding [3, 5, 6, 12, 14, 17–19], suggesting a mere temporal coincidence between the two events. These studies examined both orthodontically treated and untreated subjects, with either impacted, erupted, extracted, or congenitally absent (agenesis) third molars, some in a longitudinal, some in a cross-sectional design, and found no difference among the groups examined. Four studies report a different outcome, with an association between the presence of the third molars and the development of anterior tooth crowding [2, 13, 15, 16]. The study by Sheneman [13] was carried out on orthodontically treated subjects who presented “more stability” (probably referred to the intercanine width measurement) when the third molars were congenitally missing. However, no details are available, including statistical data, in addition to the high risk of bias of the results due to the unclear reporting of all the items examined for quality assessment of the study. Lindqvist and Thilander studied 52 orthodontically untreated subjects with impacted third molars on both sides of the mandible and then extracted the tooth on one side using the opposite side as a control [15]. Their results show that the extraction side had more favorable development of the dental arch than the control side in 70% of the cases; however, the control side had a more favorable development of the dental arch in 30% of the cases. It could be questionable to use one side of the mandible as a control, due to the fact that the two parts of the lower jaw cannot develop independently. Richardson [16] followed up two groups of patients, one with impacted third molars, the other with nonimpacted third molars, for 5 years. She found that the subjects in the former group had considerably more crowding both anteriorly and in the molar region and larger teeth than the subjects in the nonimpacted group. If it is possible that the tooth crowding is related to the presence of the impacted third molars, it is also likely that the size of the teeth had a role in creating the crowding. It must also be said that Richardson's study was associated with a high risk of bias, since it was scored “adequate” in only one item (statistical analysis). Niedzielska introduced a new element in the discussion [2]. In fact, she indicates that patients with retained third molars have higher risk of tooth crowding in relation to the Ganss ratio. Ganss ratio is the ratio between the third molar width and the retromolar space, meaning that when such space is sufficient the presence of the third molars does not cause tooth crowding; conversely, when such space is reduced the presence of the third molars can cause tooth crowding. Another theory was suggested by Al-Balkhi [20], who reported that third molars did not cause recrowding of the mandibular anterior teeth when interproximal contacts were removed. The hypothesis is that the mesial force of the erupting molars cannot be transmitted through the teeth in absence of interproximal contacts, thus preventing anterior tooth crowding. Nevertheless, the results of the present review suggest that also without removing interproximal contacts the presence or absence of the third molars does not change the outcome. Southard and coworkers [21] tested this hypothesis by studying the mesial force generated by unerupted third molars. They measured the interproximal tightness of posterior tooth contacts mesial to the second molars before and after surgical removal of the third molars on one side in 20 patients with bilaterally unerupted third molars. They found that there was a bilateral decrease in the proximal contact tightness in all contacts. These findings are in agreement with a more recent study by Okazaki [22] who investigated interproximal force change in the anterior teeth of the lower jaw and the effect of the erupting third molars in 40 treated patients. He followed them for 18 months during the retention phase. His finding also revealed that the erupting third molar did not affect the total interproximal force (Table 3).

The strength of the present review lies on the rigorous method that was followed to select and assess the studies that were included by using the PRISMA flowchart [10] and the criteria described by Vos et al. [11]. However, many of the controlled studies included obtained “inadequate” and “unclear” scoring during the quality assessment procedure (Table 2); therefore, the lack of evidence found could also be related to the low quality of the trials. Still, it must be said that the only study that scored “adequate” in all the items reported no association between the presence of third molars and anterior tooth crowding [5].

In the light of the evidence presented in this review, and based on the current findings, the presence of third molars has no significant effect and extraction to prevent anterior tooth crowding or postorthodontic relapse is not supported until proven otherwise by further well designed studies.

## 5. Conclusions

Definitive conclusions on the role of the third molars in the development of anterior tooth crowding cannot be drawn. A high risk of bias was found in most of the trials, and the outcomes were not consistent. However, since most of the studies do not support a cause-and-effect relationship between the two variables, third molar extraction to prevent anterior tooth crowding or postorthodontic relapse is not justified.

## Figures and Tables

**Table 1:** Literature review search flow chart.

Steps	Key words	Selections
1	“third molar” [MeSH] OR “third molar” [Text Word] OR “third molars” [MeSH] OR “third molars” [Text Word] OR “wisdom tooth” [MeSH] OR “wisdom tooth” [Text Word] OR “wisdom teeth” [MeSH] OR “wisdom teeth” [Text Word]	8176
2	“relapse” [MeSH] OR “relapse” [Text Word] OR “anterior crowding” [MeSH] OR “anterior crowding” [Text Word] OR “alignment” [MeSH] OR “alignment” [Text Word] OR “post retention” [MeSH] OR “post retention” [Text Word] OR “incisor relapse” [MeSH] OR “incisor relapse” [Text Word] OR “incisor crowding” [MeSH] OR “incisor crowding” [Text Word] OR “anterior post retention” [MeSH] OR “anterior post retention” [Text Word]	327628
3	Combining 1 and 2	117
4	3 limited to English and human	96
5	4 title and abstract based selection (topic, original studies)	26
6	5 references hand search	+5
7	5 + 6 controlled trials	12

**Table 2:** Quality assessment of the studies.

Author	1	2	3	4	5	6
Shanley 1962 [12]	U	U	U	U	N/A	A
Sheneman 1969 [13]	U	U	U	U	U	U
Kaplan 1974 [14]	I	A	U	A	U	A
Lindqvist and Thilander 1982 [15]	A	A	U	I	U	A
Richardson 1982 [16]	I	I	U	I	U	A
Ades et al., 1990 [17]	I	I	A	A	U	A
van der Schoot et al., 1997 [6]	I	I	U	A	U	A
Harradine et al., 1998 [5]	A	A	A	A	A	A
Little 1999 [18]	I	I	U	I	U	U
Buschang and Shulman 2003 [19]	A	A	U	A	N/A	A
Niedzielska 2005 [2]	I	A	U	A	A	A
Sidlauskas and Trakiniene 2006 [3]	I	I	U	A	N/A	U

1: sequence generation and concealed allocation; 2: size and composition of the studied groups; 3: blinding of participants, clinicians, and investigators; 4: application of inclusion and exclusion criteria for subjects; 5: descriptions of loss to follow-up; 6: adequacy of statistical analysis; A: adequate; U: unclear; I: inadequate.

**Table 3:** Summary of included studies.

Author	Study groups	Sample size	Type of study	Results
Shanley 1962 [12]	Orthodontically untreated subjects with mandibular 3rd molars: (1) bilaterally impacted (2) bilaterally erupted (3) bilaterally congenitally absent	44	Cross-sectional	No differences between the groups

Sheneman 1969 [13]	Orthodontically treated subjects with mandibular 3rd molars: in occlusion unerupted missing	49	Retrospective/longitudinal	More stability in patients with congenital missing 3rd molars than in those whose 3rd molars were present

Kaplan 1974 [14]	Orthodontically treated subjects with mandibular 3rd molars: (1) bilaterally erupted into function (2) bilaterally impacted (3) bilateral agenesis	75	Retrospective/longitudinal	No differences between the groups

Lindqvist and Thilander 1982 [15]	Orthodontically untreated subjects with mandibular 3rd molar: Extracted on one side Retained on the contralateral side	52	Prospective/longitudinal	Extraction side had a more favorable development than the control side

Richardson 1982 [16]	Orthodontically untreated subjects with mandibular 3rd molars: (1) Bilaterally impacted (2) Bilaterally nonimpacted	51	Retrospective/longitudinal	Individuals whose 3rd molars become impacted tend to have more tooth crowding

Ades et al., 1990 [17]	Orthodontically treated subjects with mandibular 3rd molars: (1) impacted (2) erupted into function (3) congenitally absent (4) Extracted at least 10 years earlier	97	Retrospective/longitudinal	No differences among the groups

van der Schoot et al., 1997 [6]	Orthodontically treated subjects with 3rd molars: (1) erupted (2) nonerupted (3) extracted (4) congenitally absent	99	Retrospective/longitudinal	No differences among the groups

Harradine et al., 1998 [5]	Orthodontically treated subjects with 3rd molars: (1) extracted (2) nonextracted	164	Prospective/longitudinal	No differences between the groups

Little 1999 [18]	Orthodontically treated subjects with mandibular 3rd molars: (1) impacted (2) erupted (3) extracted (4) agenesis	97	Retrospective/longitudinal	No differences between the groups

Buschang and Shulman 2003 [19]	Random sample of orthodontically untreated subjects as part of the Third National Health and Nutrition Examination Survey	9.044	Cross-sectional	Erupted 3rd molars not associated with increased tooth crowding

Niedzielska 2005 [2]	Orthodontically untreated subjects with mandibular 3rd molars: (1) bilaterally extracted (2) unilaterally extracted (3) bilaterally retained (4) unilaterally retained	47	Prospective/longitudinal	Retained 3rd molars associated with increased tooth crowding in relation to Ganss ratio

Sidlauskas and Trakiniene 2006 [3]	Orthodontically untreated subjects with mandibular 3rd molars: (1) erupted (2) nonerupted (3) agenesis	91	Cross-sectional	No differences between the groups
